# Procedure to categorize wheelchair cushion performance using compliant buttock models

**DOI:** 10.3389/fbioe.2022.1006767

**Published:** 2022-09-30

**Authors:** Stephen Sprigle, Yogesh Deshpande

**Affiliations:** Georgia Institute of Technology, Atlanta, GA, United States

**Keywords:** wheelchair cushion, performance, interface pressure, measurement, classification

## Abstract

**Purpose:** Wheelchair cushion prescription often seeks to address tissue integrity in addition to other clinical indicators. Because hundreds of wheelchair cushion models are available, a benefit would result if cushions were classified in a more valid manner to help guide selection by clinicians and users. The objective of this research was to develop an approach to evaluate and classify wheelchair cushion performance with respect to pressure redistribution.

**Materials and methods:** Two anatomically-based buttock models were designed consisting of an elastomeric shell that models overall buttock form and a rigid substructure that abstracts load-bearing aspects of the skeleton. Model shapes were based upon elliptical and trigonometric equations, respectively. Two performance parameters were defined, pressure magnitude and pressure redistribution. The pressure magnitude parameter compared internal pressure values of the test cushion to a flat foam reference material, resulting in three classifications, superior, comparable, and inferior. Surface sensors were used to distinguish cushions with high, moderate or low pressure redistribution performance. Ten wheelchair cushions were evaluated by both models using two loads that represent a range of body weights expected for 41–43 cm wide cushions.

**Results and Conclusion:** A classification matrix is proposed using both models and performance parameters. Two cushions met criteria for the highest level of performance, and one cushion was deemed to have inadequate performance for therapeutic value. The proposed method has a sensitivity to discern differences, compatibility with different sized cushions, and a versatility in classification. As such, it stands as an improvement over existing classification approaches.

## Introduction

Wheelchair cushions are an integral part of wheeled mobility systems. A wheelchair cushion serves both medical and functional purposes. Cushions are designed to provide wheelchair users with a support that adequately re-distributes pressure as a means to prevent pressure ulcers, supports the body in a functional posture and serves as the base for functional daily activities including transfers, weight shifts, reaching, leaning, and dynamic stability.

This combination of purposes both underscores the importance of cushion performance but also accentuates a challenge in selecting an appropriate cushion for wheelchair users. The importance of a proper seating and mobility evaluation is well documented (Davis et al., 2009; Guihan et al., 2009; Requejo et al., 2015; Chelvan and Chinduja, 2019). Seating evaluations seek to identify a cushion that provides the requisite performance to address tissue health and function during daily activities. This is a non-trivial and challenging objective, due to the differences in wheelchair users with respect to pressure ulcer risk, function and the activities they want to perform. With hundreds of different models of wheelchair cushions sold in the US, clinicians must be judicious in selecting which cushion to consider for a particular client.

For wheelchair users who are at risk for developing pressure ulcers, cushion prescription seeks to address tissue integrity in addition to other clinical indicators. Pressure magnitude has been shown to impact tissue damage in many longstanding human and animal studies (Kosiak, 1959; Dinsdale, 1974; Reswick et al., 1976; Daniel et al., 1981; Barbenel, 1991). A natural result of this research is the design of wheelchair cushions to adequately support the buttocks by either enveloping the tissues in an attempt to minimize pressure magnitudes and gradients or redistributing forces away from tissues under bony prominences. For these cushions, performance can be reflected by their ability to manage and redistribute pressure on the buttocks. Over the years, cushion performance has been evaluated using both human subjects and buttock model or phantom testing.

Multiple studies have used human subjects to assess pressure redistribution performance (Swain and Peters, 1997; Kernozek and Lewin, 1998; Burns and Betz, 1999; Ferrarin et al., 2000; Ingrid Eitzen et al., 2003; Gil-Agudo et al., 2009; Hollington and Hillman, 2013; Lee et al., 2016; Kawasaki et al., 2021). Use of wheelchair users offers the most ecologically valid means to assess load distribution but is burdened with a few key limitations. These limitations can be illustrated by two recent reviews of studies comparing wheelchair cushion performance (He and Shi, 2020; Damiao and Gentry, 2021). When combined, results were not terribly surprising: relative cushion performance depended on the metrics used to define performance, the cushions included in the comparisons, and the cohort of human subjects. In other words, comparing many cushions using human subjects is simply not feasible.

Cushion pressure redistribution performance has also been investigated using indenters and buttock models (Chow and Odell, 1978; Reddy et al., 1982; Sprigle et al., 2003; Pipkin and Sprigle, 2008; Iizaka et al., 2009; Akins et al., 2011; Paul et al., 2013; Hollington et al., 2014; Kumar et al., 2015). Bench tests are typically more repeatable and sensitive than human subject testing. Bench testing is the desired approach of standards granting bodies since test methods must be reliable and valid when performed by different people or organizations in different locations. Two standards-granting bodies, the International Standards Organization and RESNA, have developed a series of standards focused on testing wheelchair cushion performance relative to their abilities to manage tissue integrity (International Organization for Standardization (ISO), 2018; ANSI/RESNA WC-3:2018, 2018). The test methods address many constructs, but none directly measure a cushion’s performance in managing and re-distributing loads on the body.

The ability to compare and contrast wheelchair cushion performance is important from both clinical and policy perspectives. To support seating evaluations, clinicians would benefit from knowing categories of cushion that offer performance relative to a standard material. This would allow clinicians to narrow possibilities to a manageable number while assessing other cushion features and characteristics to best meet the needs of the user. Clinical experience and multiple studies (Shaw, 1993; Ingrid Eitzen et al., 2003; Stockton and Rithalia, 2009) consistently assert that no one cushion is best for everyone and cushion prescription is “highly individual” (Ingrid Eitzen et al., 2003).

Cushion performance is also important from a policy or reimbursement perspective. In the United States, wheelchair cushions are classified using HCPCS codes with each classification linked to a reimbursement level. Commercial products are classified using 12 codes, defining 6 cushion categories with each presented in two size groupings. Three categories are defined which are based upon criterion meant to reflect performance using the terms general use, positioning, and skin protection. Skin protection cushions are provided to persons with a “current pressure ulcer or past history of a pressure ulcer on the area of contact with the seating surface” or “absent or impaired sensation in the area of contact with the seating surface or inability to carry out a functional weight shift” (Center for Medicare and Medicaid Services, 2019) . Inherent to this coverage policy is the implication that skin protection cushions offer a higher level of pressure management compare to other categories. Four categories include the term “skin protection” descriptors because some also meet the criterion as positioning cushions (HCPCS codes E2603/4, E2607/8, E2622/3, E2624/5).

Classification should strive to assign the same code to cushions with similar pressure management performance. However, the current method to classify cushions is based upon a test of immersion (PDAC, 2004). This test loads an indenter to determine how far it immerses into the cushion and was designed to describe cushion characteristics not to reflect pressure management performance (Sprigle et al., 2001).

The result of this classification system is that codes include cushions with disparate performance. For example, the Pricing, Data Analysis and Coding Contractor (PDAC) lists over 500 cushion models with a “skin protection” designation, highlighting the vast array of products in this category.

A benefit would result if cushions were classified in a more valid manner for use by clinicians and users as well as manufacturers. The objective of this research was to develop a procedure to assess wheelchair cushion performance with respect to their pressure management performance that can be used to categorize cushions. The intent to propose a new categorization scheme is based, in part, on the limitations of the current system.

## Materials and methods

The overall premise of the test procedure is to evaluate cushions using two different compliant buttock models instrumented to measure pressures internally and on their surface. These models are configured using a rigid substructure surrounded by a compliant elastomer that serve as analogs to the bony skeleton and soft tissue surrounding the buttocks.

The buttock model shapes reflect abstractions of buttock shapes and were inspired by data of cushion contours under load (Sprigle and Schuch, 1993; Brienza et al., 1999), clinical experience measuring interface pressures as well as reflections of the anatomy of the pelvis and femurs. Additionally, they reflected the design objective of creating two distinct shapes. One model has a rounded or curved shape with the other being more peaked to highlight the bony skeleton. A full description of the design and validation of one model has been reported elsewhere but will be briefly described here (Kumar et al., 2015).

**FIGURE 1:**
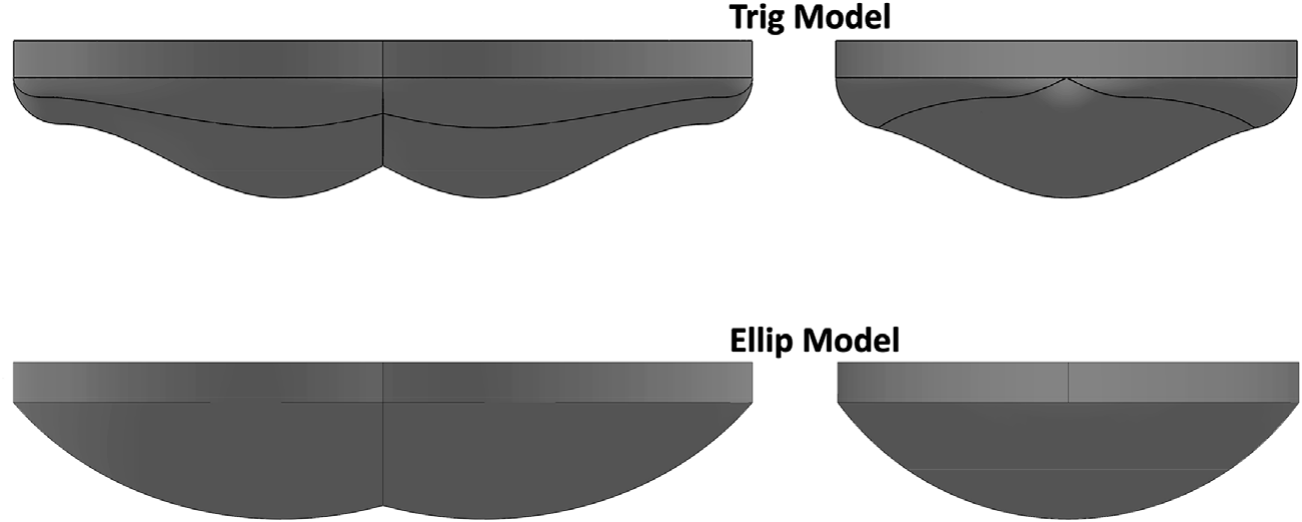
Shapes of elliptical and trigonometric elastomer shells.

The outer compliant elastomeric shells of both models were fabricated using the same elastomer (Dragon Skin™ silicone, Smooth On, Inc.) and have the same overall width. Two outer shells were designed that differ in profile, with both being parametrically based on elliptical (ellip model) and trigonometric (trig model) equations. The parametric design permits scaling so the overall form can be applied to create models appropriate for larger and smaller cushion widths. This project used the 40 cm wide model designed for cushions with a 41–43 cm width. The substructures are positioned within the compliant model to have 2 cm of elastomer beneath the medial protuberances of the rigid substructure. Figure 1shows two views of each model highlighting the differences in form.

**FIGURE 2:**
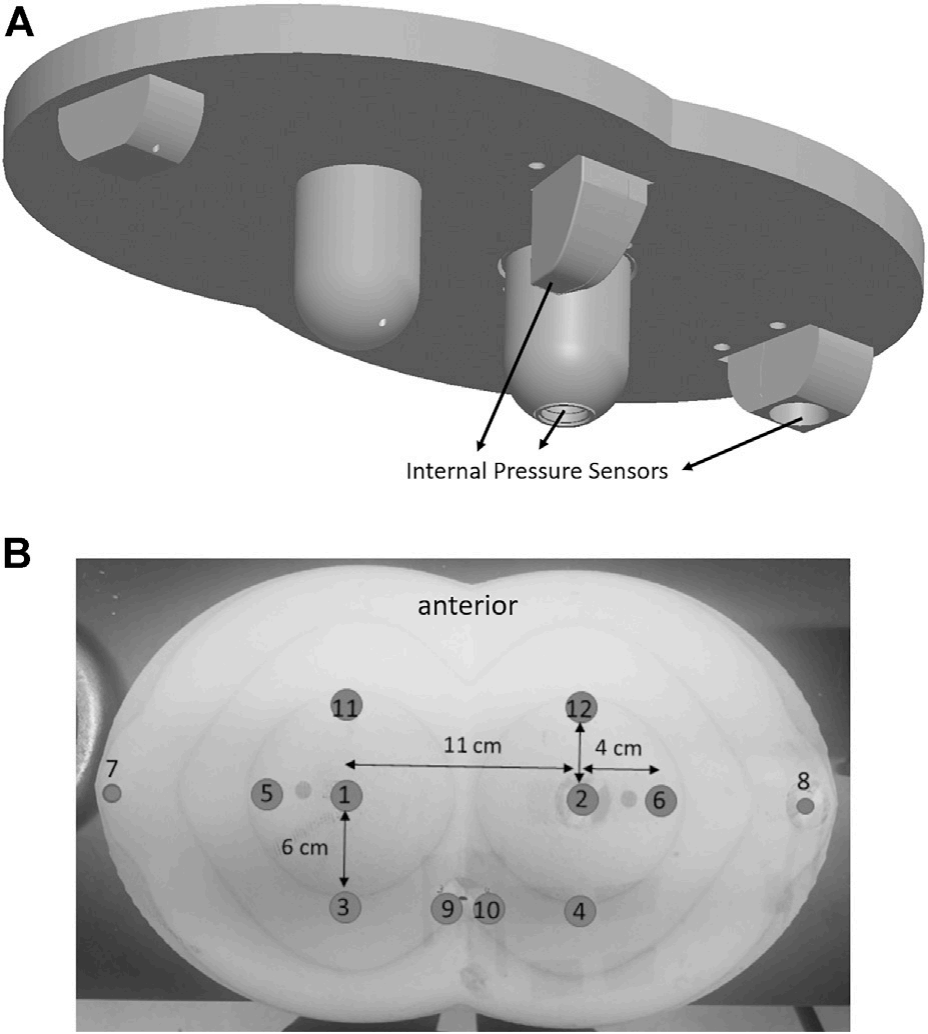
**(A)** Rigid substructure, internal pressure sensor locations and **(B)** surface sensor locations depicted on the trigonometric model.

The rigid substructure was fabricated to reflect the anatomy of the load-bearing aspects of the pelvis and femurs. This abstraction of the anatomy results in five protuberances that serve as analogs for the ischial tuberosities, trochanters, and coccyx (Figure 2A).

### Instrumentation

Two elements of the substructure are instrumented with 19 mm diameter pressure sensors (TD10 model, STS AG, Sirnach, CH) to measure internal pressures at the substructure-elastomer interface. The sensors were calibrated to 350 mmHg using a pneumatic calibration rig. The coccyx analog protuberance was instrumented with a Flexiforce sensor (Tekscan, model A201) that is described below.

In addition, 12 force sensors (A201 and A401 Flexiforce, Tekscan, Boston) are placed at 6 axisymmetric locations over the surface. Ten sensors (A401) have a 0.875 in sensing diameter with the two sensors placed underneath the lateral protuberances (A201) having a 0.3125 in sensing diameter. Surface sensors were covered by a thin adhesive-based film (Con-Tack Brand, Pomona, CA), This shim helps isolate the sensor layers from surface forces and is the preferred practice as defined by the manufacturer (Tekscan, 2020). Each were calibrated prior to about 455 mmHg using a bench-top jig that steps through forces over the defined range.

Surface sensor placement is divided between locations under rigid substructure protuberances (i.e., bony) and regions outside of the substructure (i.e., nonbony). Specifically, sensors 1 and 2 lie below the medial protuberances, sensors 7 and 8 lie under the lateral, and sensors 9 and 10 lie under the medio-posterior protuberance (Figure 2). The remaining 6 sensors are placed forward (11 and 12), lateral (7 and 8) and rearward (3 and 4) to the medial protuberance. Dual axisymmetric locations are used for a better estimate of the true value compared to using a single location.

The elastomeric shell was covered by a thin cotton stockinette. This was done to standardize the model surface that contacts the cushion in order to eliminate any potential surface differences of the elastomer, which can be tacky. Surface sensors were affixed to the model using double-sided tape at their respective locations.

### Performance parameters

Wheelchair cushion pressure management performance is characterized by 2 parameters which reflect different aspects of performance, pressure magnitude, and pressure redistribution. Pressure magnitude is measured using the internal sensors with pressure redistribution measured using the surface sensors.

### Testing procedure

Cushions are loaded with the buttocks model using a mechanical testing machine or loading apparatus. After two conditioning loads, the test load is applied and held for 90 s after which pressure measurements are recorded at 1 Hz. Measurements are taken at two different loads, 50 and 60 kg for 41–43 cm wide cushions to simulate a range of user mass. These masses reflect the estimated buttocks loading of persons with body mass of 76 and 92 kg, which represents the 50th and 84th percentile body mass of adults (SAE International, 2003) who have seated hip widths between 38 and 43 cm. This hip width range was defined to reflect the size of persons who would likely be prescribed 41–43 cm wide cushions. The rationale of using two loading parameters is intuitive, namely, people with different body mass use the same size cushion, so testing should reflect a range of body mass. As with the models, these loading parameters can be scaled to reflect body masses of persons using larger and smaller sized cushions.

### Surface pressure redistribution measurement and analysis

The pressure redistribution parameter uses surface sensors to calculate the percentage of surface pressures at locations under the 3 rigid protuberances (SumBony) relative to the sum of all surface sensors (TotSurfSum). Referencing Figure 2,

$$\begin{array}{c} \%Bony=\frac{SumBony}{TotSufSum}=\frac{Sum\;of\;values\;at\;sensors\;1,2,7,8,9,10}{Sum\;of\;values\;1-12} \end{array}$$ (1)

An enviable cushion should redistribute pressures away from high risk areas under bony prominences. Consider sitting on a rigid or low compliance surface. The majority of pressures will be borne by bony aspects rather than being redistributed over the buttocks surface, representing poor pressure redistribution.

The mean and standard deviation of the %Bony parameter was calculated using 6 repeated trials on each cushion and at each load. Analysis is based upon the 95th percentile confidence interval (CI):

CI=μ±σn (2)

where *µ* = sample mean; *σ* = sample standard deviation, and *n* = number of trials.

Combining the results of the two loading profiles purposely impacts the analysis because the CIs will be larger due to increased variance. A cushion which supports the two loads in a similar manner will benefit by a resulting smaller CI. This is fair, because cushions that support persons of different mass in a similar manner reflect an advantageous performance.

Thresholds were defined using a combination of a theoretical premise and empirical results. The redistribution parameter is a criterion-referenced value using 50% for the ellip model, thus establishing the upper threshold that 50% of the surface pressures are under “bony” areas, which reflects a pressure redistribution away from the high-risk areas. The trig model uses a threshold of 55%, reflecting a theoretical premise that its design with a more prominent curvature and peaked shape results in greater loading under the medial protuberance. In addition, empirical testing of a 3” block of high resiliency foam with a 44 ILD produced %Bony values of 0.5 using the ellip model and 0.56 on the trig model.

The criteria are defensible based upon the benefit of redistributing pressures away from the underlying bony protuberances of the models. This parameter will reflect the performance of off-loading cushions which redirect pressures away from at-risk sites as well as enveloping cushions which seek to equalize pressures across the buttocks. As such, greater redistribution reflects a more desirable performance category regardless of the approach adopted by the cushion.

The redistribution parameter is used to create a trichotomous classification, being divided by whether the 95th % CI falls below, around, or above the respective threshold, using high, moderate and low categories (Table 1).

**TABLE 1:** Pressure redistribution classification definitions.

Designation	Elliptical criterion	Trigonometric criterion	Description
High redistribution	<0.5	<0.55	The CI falls below criterion
Moderate redistribution	∼0.5	∼0.55	The CI includes criterion
Low redistribution	>0.5	>0.55	The CI lies above criterion

### Internal pressure measurement and analysis using equivalence testing

Internal pressures offer an opportunity to represent the loading that occurs at the tips of bony prominences. Longstanding research suggests that soft tissue stresses are the greatest at the interface between bone and soft tissues (Bennett, 1973; Chow and Odell, 1978; Zhang et al., 1997; Bouten et al., 2003).

The parameter of interest is the sum of the three internal sensors (SumInt) within the model substructure. This evaluation uses a reference material to determine performance. An advantageous cushion will have lower SumInt values than the reference material. Reference materials consist of flat block foam with different references used for Skin Protection and General Use designations. When evaluating Skin Protection cushions, a block of 3” thick high resiliency (HR) foam is used with a nominal IFD value near 45 lb and a density exceeding 2.5 lb/in3. General Use cushions are evaluated against a 2” HR45 HR foam block. This creates a more stringent standard for Skin Protection cushions compared to General Use cushions, reflecting the intent of the HCPCS classifications.

Equivalence tests assess when device performance differs by more than a practically relevant or meaningful amount. Its analysis is often more appropriate than inferring a lack of a difference when assessed by traditional statistical means (Wellek, 2010). Operationally, equivalence tests are a two-sided evaluation of differences using confidence intervals. A formal equivalence test is not being proposed here, rather its underlying premise and computational approach is be adopted to compare a Reference material to Test cushions.

Test Mean/Reference Mean is the parameter of interest, including the CI of this ratio. Its relationship to pre-defined equivalence limits are used to assign three categories of pressure magnitude performance. Lower magnitude of pressures exhibited by the Test cushion indicates a higher level of performance (Table 2).

**TABLE 2:** Pressure magnitude classification definitions.

CI_ρ_ ≤ LEL	Superior. The entire CI of the ratio (*ρ*) of the mean of the test cushion to the mean of the reference material is less than or equal to the lower equivalence limit (LEL)
CI_ρ_ ≥ UEL	Inferior. The entire CI of the ratio (*ρ*) of the mean of the test cushion to the mean of the reference material is greater than or equal to the upper equivalence limit (UEL)
Low CI_ρ_ > LEL or high CI_ρ_ < UEL	Comparable. The CI of the ratio (*ρ*) of the mean of the test cushion to the mean of the reference material crosses either the LEL or UEL or lies fully between them

LEL, lower equivalence limit (0.9); UEL, upper equivalence limit (1.1); “1’”, unity line.

The lower equivalence limit is set equal to 0.9 and the upper equivalence limit = 1.1 which define a noticeable or meaningful difference at 10%. To define the equivalence limits, a literature search was undertaken to identify interface pressure data collected on different cushions. Interface pressure data was extracted that reported the mean and standard deviation of localized pressures, such as the Peak Pressure Index (Sprigle et al., 2001). The dataset was obtained from two articles (Brienza et al., 2001; Crane et al., 2016) in addition to data collected in the REAR Lab at Georgia Tech. In total, interface pressure data on nine cushions were included, spanning 62 persons, in total. The standard deviation and subject number were used to calculate 1.96*SEM. The ratio of 1.96*SEM/Mean was calculated and averaged over all the cushions to gain an estimate of the size of a CI normalized to the mean. The average ratio was 0.153. Because IPM measured on human subjects is considered to have more variance than data taken using a buttocks model, an EL = 0.1 was defined.

**FIGURE 3:**
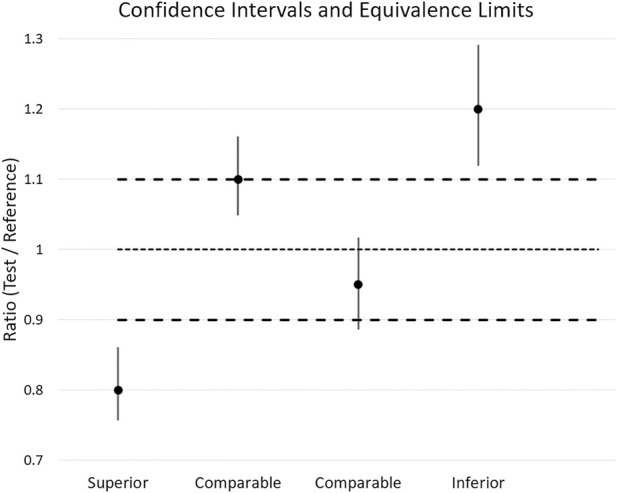
Examples of classifications based upon point estimates and CI relative to ELs.

Analysis: To calculate the needed parameters (estimate of the mean ratio and its CI), a two-sided equivalence test can be applied, or else the required parameters can be computed manually. To illustrate the classification approach, sham data was used to create examples of cushions with varying ratios and CIs (Figure 3). The Superior example is indicated by the CI falling below the 0.9 LEL, signifying superior performance. In distinction, the inferior CI lies above the 1.1 UEL. These two conditions indicate a significant difference compared to the reference material. Two “comparable” examples are also depicted in which the CI crosses the LEL and UEL.

### Cushion cohort

A cohort of 10 commercial wheelchair cushions were evaluated (Table 3). Six cushions had been classified with a Skin Protection designation and three as General Use (GU) wheelchair cushions. These cushions were selected because they vary in design and material construction. Cushions with a Skin Protection designation were evaluated against the 3” HR45 foam reference and the GU cushions were evaluated against the 2” HR45 foam reference. Using the current HCPCS classifications, the 3” foam reference would meet the Skin Protection criteria and the 2” reference material would meet the GU category requirements.

**TABLE 3:** Cushion cohort.

Cushion model	HCPCS code	Classification	Manufacturer	City	State
Jay 2	E2622	Adj skin pro/pos	Sunrise Medical	Longmont	CO
Matrx Vi	E2607	Skin pro/pos	Motion Concepts	Concord	Ontario
ActaEmbrace	E2607	Skin pro/pos	Comfort Company (Permobil)	New Berlin	WI
Ride forward	E2607	Skin pro/pos	Ride Designs	Littleton	CO
Gel pro elite	E2603	Skin pro	Blue chip medical products	Suffern	NY
GeoMatt PRT	E2603	Skin pro	Span America Medical Systems	Greenville	SC
CrossCut	E2601	General use cushion	Span America Medical Systems	Greenville	SC
Amara 100	E2601	General use cushion	Blue Chip Medical Products	Suffern	NY
Medline gel	E2601	General use cushion	Medline Industries	Northfield	IL
Gel-U-seat lite	E2601	General use cushion	Drive Medical	Port Washington	NY

Adj, adjustable; Pro, protection; Pos, positioning.

## Results

### Pressure redistribution

Pressure redistribution uses a criterion-referenced classification and all three classifications were reflected in the results (Table 4). Skin Protection cushions were classified as having high or moderate redistribution performance on the ellip model but 4 cushions were classified as having low redistribution on the trig model. Both the Jay 2 and Ride Forward cushions exhibited high redistribution on both models.

**TABLE 4:** Cushion classification based upon pressure redistribution.

Model	Category	Cushion	Mean	CI+	CI-	Redistribution
Ellip	Skin Pro	ActaEmbrace	0.50	0.50	0.49	Moderate
		GelProElite	0.50	0.51	0.49	Moderate
		GeoMattPRT	0.47	0.48	0.47	High
		J2	0.46	0.47	0.45	High
		MatrxVI	0.50	0.51	0.49	Moderate
		Ride Forward	0.42	0.42	0.41	High
					
	GU	Amara100	0.55	0.56	0.55	Low
		CrossCut	0.53	0.54	0.53	Low
		Gel-U-Seat Lite	0.52	0.52	0.51	Low
		Medline Gel	0.50	0.51	0.49	Moderate
					
	Reference	HR44 3in	0.50	0.50	0.49	Moderate
		HR44 2in	0.52	0.53	0.51	Low
Trig	Skin Pro	ActaEmbrace	0.58	0.58	0.57	Low
		GelProElite	0.57	0.58	0.56	Low
		GeoMattPRT	0.60	0.61	0.59	Low
		J2	0.53	0.54	0.53	High
		MatrxVI	0.57	0.58	0.57	Low
		Ride Forward	0.52	0.53	0.52	High
					
	GU	Amara100	0.60	0.61	0.59	Low
		CrossCut	0.67	0.69	0.65	Low
		Gel-U-Seat Lite	0.54	0.54	0.53	High
		Medline Gel	0.69	0.71	0.68	Low
					
	Reference	HR44 3in	0.57	0.57	0.56	Low
		HR44_2in	0.64	0.66	0.63	Low

The majority of GU cushions reflected low pressure redistribution. This is not surprising because these cushions tend to have less thickness, which is needed to adequately redistribute pressures upon deformation. Two exceptions are noted by the Medline Gel on the ellip model which exhibited moderate redistribution and Gel-U-Seat Lite on the trig model which had high redistribution.

The tables also list the redistribution classifications of the two reference materials using internal pressure magnitude assessment. This simply offers some orientation as to how these materials redistribute pressures.

The CI ranges of the parameter were fairly tight across the cushions despite being reflective of values measured using two applied loads.

### Pressure magnitude

Classifications of the Skin Protection cushions using the SumInt pressure parameter is assigned against the 3’’ reference material. Pressure magnitude classifications revealed both “superior” and “comparable” performance (Table 5). Moreover, the classifications were consistent across the two models. No cushion was found to have inferior pressure magnitude.

**TABLE 5:** Pressure Magnitude classifications of Skin Protection cushions.

Model	Cushion	Mean	CI+	CI-	Classification
Ellip	ActaEmbrace	0.83	0.89	0.77	Superior
	GelPro Elite	0.84	0.91	0.78	Comparable
	GeoMatt PRT	0.97	1.04	0.90	Comparable
	Jay 2	0.80	0.86	0.75	Superior
	Matrx Vi	0.87	0.93	0.81	Comparable
	Ride Forward	0.70	0.75	0.66	Superior
Trig	ActaEmbrace	0.84	0.88	0.80	Superior
	GelPro Elite	0.98	1.04	0.92	Comparable
	GeoMattPRT	0.95	1.01	0.89	Comparable
	J2	0.78	0.82	0.74	Superior
	MatrxVi	0.96	1.01	0.91	Comparable
	Ride Forward	0.75	0.78	0.71	Superior

Pressure magnitude performance of GU cushions resulted in all three classifications being assigned when referenced to the 2” reference material (Table 6). One cushion, the Medline Gel, was assigned an inferior classification on both models. The Gel-U -Seat Lite cushion was defined as having comparable magnitude on the ellip model but superior performance on the trig model.

**TABLE 6:** Pressure magnitude classifications of General Use cushions.

Model	Cushion	Mean	CI+	CI-	Classification
Ellip	Amara100	0.97	1.04	0.91	Comparable
	CrossCut	1.14	1.23	1.06	Comparable
	Gel-U-Seat Lite	0.95	1.02	0.89	Comparable
	Medline Gel	1.20	1.29	1.11	Inferior
Trig	Amara100	0.92	0.97	0.87	Comparable
	CrossCut	1.03	1.10	0.96	Comparable
	Gel-U-Seat Lite	0.74	0.78	0.70	Superior
	Medline Gel	1.18	1.25	1.11	Inferior

### Repeatability

Pressure parameters were calculated based upon six trials performed at each load with each model. Repeatability of the parameters were assessed using the coefficient of variation (Table 7). The variation across repeated trials reflect the differences in reliability of the internal and surface sensors within their respective contexts of use. Overall, the results indicate a high repeatability across the trials.

**TABLE 7:** Repeatability of pressure parameters.

Parameter	Load	Model	CV
SumInt	50	Ellip	<1%
		Trig	<1%
	60	Ellip	<3%
		Trig	<4%
TotSurf	50	Ellip	<4.5%
		Trig	<5%
	60	Ellip	<6%
		Trig	<7%

## Discussion

Overall, the testing procedure reflects several decisions that were made to create a more valid means to measure wheelchair cushion performance: use of two compliant indenters, use of multiple load profiles, and use of two parameters to reflect performance. The models are parametrically designed so that they can be scaled to evaluate different sized cushions. This is important since cushions with greater size are designed for persons with greater body mass that should be reflected in testing.

The use of a compliant model is definitely more complex than using a rigid indenter. But a compliant indenter with a rigid substructure has key advantages. Compliant models have greater validity in representing the human buttocks so, the added complexity is a defensible decision. The use of a compliant model permits pressure measurement using internal sensors mounted on a rigid substructure. This is a more valid representation of loading on the tissues compared to using surface sensors mounted to a rigid indenter. Using two models provides a fuller evaluation of performance that reflect different body types. The ellip model reflects persons with fairly typical buttocks tissue mass whereas the trig model reflects persons with lesser tissue mass due to atrophy, cachexia or other causes. Persons with both body types can be at risk of ulceration, so performance testing should consider a range.

Wheelchair cushions are used by persons who have a range of body masses, so cushion performance is best measured in a manner that reflects this range. This test used two loads that are based upon a range of human masses of persons with hip widths that would fit the size of the evaluated cushion. Performance parameters are calculated from trials run at both loads, thereby reflecting the average performance at the two loads. A cushion that offers similar performance to load over this range would be a more enviable cushion and the test results would reflect that functionality.

Two parameters are designed to reflect meaningful differences across the wide array of cushion designs. In a general sense, wheelchair cushions adopt two main approaches, off-loading and envelopment. Enveloping cushions seek to equalize pressures over the buttocks surface whereas off-loading cushions seek to re-direct pressures away from at-risk sites. Indeed, some cushions seek a combination of both approaches. A test method should not be biased to one approach. Rather, this method seeks to reflect the underlying purpose of wheelchair cushions, namely pressure management.

The pressure redistribution parameter applies a criterion-referenced test that uses absolute thresholds. This parameter reflects a cushion’s ability to re-distribute forces away from at-risk areas, namely bony prominences. This parameter reports the percentage of pressures under the substructure elements. For the ellip model, a threshold of 50% is defined with the trig model having a criterion of 55%, with the evaluation being tied to a confidence interval. The pressure redistribution parameter does not report pressure magnitudes. Not including pressure magnitude of surface sensors was based on the fact that the pressure redistribution parameter is used in combination with the pressure magnitude that reflects magnitude.

The pressure magnitude parameter also uses a criterion-referenced test but uses thresholds tied to a reference material. Two reference materials were selected that represent a minimum level of performance for different cushion categories, namely general use and skin protection. Testing permits evaluation of whether the test cushion offers superior, comparable or inferior performance based on a mean value with its confidence interval.

Therefore, cushion performance is evaluated using two synergistic parameters that utilize confidence intervals. This addresses the difficulty in applying thresholds to define cushion categories. The use of a single point estimate, such as the mean, does not reflect the uncertainty of that estimate. A benefit exists from using both the point estimate and dispersion in the analysis. The use of a CI provides the best estimate of the true value of the point estimate or mean. However, another practical benefit exists with this approach. Testing included 6 repeated trials at two loads or a total of 12 trials to calculate the mean and CI. If cushion testing results in a borderline case, it can serve as motivation for additional testing. Adding trials to the test has the potential to reduce the CI and to gain a better estimate of the true mean; afterward, the results can be re-assessed. This may be particularly important in cases where the CI minimally crosses a threshold value.

For example, referring to Table 5, the GelPro Elite has a mean pressure magnitude of 0.84 and CI of 0.78–0.91. This CI minimally crosses the Lower Equivalence Limit of 0.9, resulting in a “comparable” classification. The manufacturer would then have the option of adding more trials to the test method in hopes of reducing the CI which may position the cushion in the “superior” classification. Similarly, tests of pressure redistribution (Table 4), identifies the ActaEmbrace at being right on the border of moderate versus high redistribution. This can trigger additional trials as a means to gain a better estimate of the true mean and variance.

A few possible outcomes can be identified from the comparisons of GU cushions with the 2” reference. Note that the Gel-U-Seat Lite had superior performance relative to the 2” reference. This result should trigger comparison with the 3” HR45 reference, because the Gel-U-Seat Lite may, in fact, be comparable to it. Relatedly, the Medline Gel cushion was deemed to have inferior performance. This may be interpreted to mean that this cushion does not have requisite performance to warrant reimbursement as a therapeutic product and may motivate the manufacturer to improve its design.

Based on the results, internal pressure magnitudes at the three substructure locations were disparate, with the highest pressures being found at the medial protuberance and the lowest under the coccyx analog. This was not unexpected and reflects the interface pressure distributions measured on seated humans. However, the design of the model that abstracts these 5 bony areas of the skeleton has value from a larger perspective. Some persons may want to evaluate cushions against postural asymmetries. These model configurations can be used to model pelvic obliquity or pelvic tilt simply by mounting the model with slight rotation. These two postures are fairly common in wheelchair users and result in different protuberances playing more prominent load-bearing roles.

The use of two models at two loads also allows proper evaluation of cushions deemed to be “adjustable”. The current system does not require cushions to be adjustable relative to performance, rather it limits this construct to fluid-based cushions using immersion (Center for Medicare and Medicaid Services, 2019). In distinction, this test procedure allows for adjustability to be evaluated by pressure redistributing performance regardless of material construction using two models and/or two loads, resulting in a more valid classification.

### Proposed classification system

The results of this cushion evaluation procedure can be used to propose a more robust system to improve cushion classification based upon pressure management performance.

The proposed classification would create four cushion categories, highlighted by 3 levels of pressure management (Table 8). A top-level cushion would have to meet very high criteria, requiring high performance on both parameters as evaluated with both elliptical and trigonometric models. Only a small number of cushions would be expected to meet these criteria and would be deemed as appropriate options for persons with the highest levels of risk. In the tested cohort, only the J2 and Ride Forward cushion met this top-level criterion. A mid-level category would require cushion to reflect moderate redistribution and comparable pressure magnitude performance against reference. Because Level 2 requires good performance using both models, they would serve as appropriate options for a wide range of users. Finally, a lower level pressure management cushion would use only the ellip model, and require moderate redistribution and comparable pressure magnitude performance to the 3” reference. These cushions would be more appropriate for users who have fairly typical buttock tissue bulk, but are still at risk of tissue damage. A General Use category would include cushions that have therapeutic value for wheelchair users who do not require pressure management but still require proper seating support.

**TABLE 8:** Proposed wheelchair cushion classification matrix.

	Pressure redistribution	Pressure magnitude
1st level pressure management	“high” classification on both ellip and trig models	Superior classifications on both ellip and trig models against 3” reference
2nd level pressure management	At least “moderate” classification on both ellip and trig models	At least “comparable” classifications on both ellip and trig models against 3” reference
3rd level pressure management	At least “moderate” classification on ellip model	At least comparable classification using ellip model against 3” reference
General use cushion		At least comparable classification using ellip model against 2” reference
Cushions not meeting minimum performance requirements		“Inferior” classification using ellip model against 2” reference

A minimum level performance level would be required for a cushion to be deemed therapeutic. Cushions found to be “inferior” to the 2” reference cushion in pressure magnitude would not quality as wheelchair cushions. This would require manufacturers to redesign the cushion to have therapeutic benefit. This, then, defines a 2” block of HR45 foam as the *de facto* level of minimal therapeutic benefit.

The use of dual parameters to reflect pressure management performance allows for the definition of alternative classification systems. For example, a two-tier system could be defined by lowering the minimal requirements.

### Limitations and future effort

The described test procedure was evaluated for reliability using 10 cushions. While these cushions spanned a wide range of material design and construction, a larger cohort may have identified undesirable interactions between cushion design and the test method. Relatedly, only one cushion was classified as being “adjustable” (J2). It was not adjusted during testing but still reported high performance. Evaluation of the test protocol using a cohort of adjustable cushions is needed to insure the test is sensitive to certain adjustments. That being said, in the U.S, the current moniker of ‘adjustable’ is assigned to cushions without performance testing, so not all products with said designation may actually reflect adjustable performance relative to pressure management.

Test methods must be defined to measure cushions with greater size. In the US, four size models and accompanying test methods should be developed for cushions with widths of 36–38 cm, 41–43 cm, 46–48 cm, and 55 + cm. As stated, because the compliant models are parametrically designed, the test method described here can be scaled to other size models and loading parameters.

The use of surface sensors can result in erroneous data on cushions with discontinuous loading surfaces. This is a random occurrence based upon how the cushion deflects and deforms under load. In this study, one cushion exhibited discontinuous surface, the Jay2, a fluid bladder that can have invaginations of its fluid bladder under load. During testing, 4 out of 288 datapoints were flagged as outliers.

In this study, an outlier was defined as a datapoint that differs from the median value by 50% or more within a model-load condition. The use of a median was deemed advantageous over parametric analysis (such as Z-score) due to the 6 trials per condition. Because each buttock location is represented by 2 sensors spaced asymmetrically, the removal of an outlier did not obviate the use to the entire trial. The low number of outliers identified during testing offers some evidence that the use of surface sensors can report valid data on cushions with discontinuous surfaces, although investigating other cushions that exhibit discontinuous surfaces, such as air cell cushions and the Vicair, is warranted. Adding trials to the test method will be one means to manage outliers if further testing indicates a need.

The life of compliant models should be defined as a part of the test method. All compliant materials can degrade over time. Within this study, data was collected over a few weeks, so mechanical changes were not an issue. Over a longer timeframe, the elastomer’s material properties of the two compliant indenters elastomer were measured over an 18-month period and showed no change in stiffness. Relatedly, the test should define how often sensors should be re-calibrated. The surface sensors and internal sensors reflect markedly different transducer designs and will probably have to be re-calibrated at different frequency. Based upon calibration checks, on average, surface sensors should be calibrated every 3 weeks with internal sensors needing re-calibration after about 8 weeks. One benefit of using a reference material when calculating pressure magnitude parameters is that small changes in calibration or elastomeric properties will not impact the test results compared to absolute parameters.

## Conclusion

A test procedure to evaluate wheelchair cushion performance is proposed to better evaluate and classify cushions as a means to better inform clinicians and users. During the development and finalization of this test procedure to measure cushion performance, several aspects of validity were considered and demonstrated. The procedure uses parametrically designed compliant models that can be scaled to evaluate different sized cushions. The construct of pressure management performance is reflected by synergistic parameters that reflect both the magnitude of pressures at specific regions of interest and the ability of the cushion to redistribute pressures relative to those locations. These parameters were based upon the longstanding knowledge that tissue damage tends to occur in the areas under bony prominences. These parameters are calculated under two testing loads that are based upon the sizes of persons who would fit on the tested products, as a means to measure performance in a realistic manner. Classification is performed using the confidence intervals of these distributions against defined criteria. The results indicate that the parameters are measured in a repeatable manner and classification is able to distinguish wheelchair performance across cushion models. Repeatability and sensitivity to detect differences are methods to assess validity of measurements. Classification of cushion performance can be adjusted across a range of rigor. A clinical value exists to utilize multiple performance categories, but other factors or stakeholders might indicate a need to use less rigorous classification. In summary, the proposed method has a sensitivity to discern differences, compatibility to be used with different sized cushions, and a versatility in classification. It thus stands as an improvement over the existing classification system.

## Data Availability

The raw data supporting the conclusion of this article is available at https://smartech.gatech.edu/handle/1853/54079 without undue reservation.
